# Spatial and space–time distribution of *Plasmodium vivax* and *Plasmodium falciparum* malaria in China, 2005–2014

**DOI:** 10.1186/s12936-016-1646-2

**Published:** 2016-12-19

**Authors:** Samuel H. Hundessa, Gail Williams, Shanshan Li, Jinpeng Guo, Linping Chen, Wenyi Zhang, Yuming Guo

**Affiliations:** 1 Division of Epidemiology and Biostatistics, School of Public Health, University of Queensland, Herston Rd, Herston, QLD 4006 Australia; 2 Institute for Disease Control and Prevention, Academy of Military Medical Science, Beijing, People’s Republic of China

**Keywords:** *Plasmodium falciparum*, *Plasmodium vivax*, Malaria, Spatial clustering, Space–time clustering

## Abstract

**Background:**

Despite the declining burden of malaria in China, the disease remains a significant public health problem with periodic outbreaks and spatial variation across the country. A better understanding of the spatial and temporal characteristics of malaria is essential for consolidating the disease control and elimination programme. This study aims to understand the spatial and spatiotemporal distribution of *Plasmodium vivax* and *Plasmodium falciparum* malaria in China during 2005–2009.

**Methods:**

Global Moran’s *I* statistics was used to detect a spatial distribution of local *P. falciparum* and *P. vivax* malaria at the county level. Spatial and space–time scan statistics were applied to detect spatial and spatiotemporal clusters, respectively.

**Results:**

Both *P. vivax* and *P. falciparum* malaria showed spatial autocorrelation. The most likely spatial cluster of *P. vivax* was detected in northern Anhui province between 2005 and 2009, and western Yunnan province between 2010 and 2014. For *P. falciparum,* the clusters included several counties of western Yunnan province from 2005 to 2011, Guangxi from 2012 to 2013, and Anhui in 2014. The most likely space–time clusters of *P. vivax* malaria and *P. falciparum* malaria were detected in northern Anhui province and western Yunnan province, respectively, during 2005–2009.

**Conclusion:**

The spatial and space–time cluster analysis identified high-risk areas and periods for both *P. vivax* and *P. falciparum* malaria. Both malaria types showed significant spatial and spatiotemporal variations. Contrary to *P. vivax*, the high-risk areas for *P. falciparum* malaria shifted from the west to the east of China. Further studies are required to examine the spatial changes in risk of malaria transmission and identify the underlying causes of elevated risk in the high-risk areas.

**Electronic supplementary material:**

The online version of this article (doi:10.1186/s12936-016-1646-2) contains supplementary material, which is available to authorized users.

## Background

Malaria is a life-threatening infectious disease severely affecting vulnerable communities in tropical and subtropical regions where the environment is suitable for transmission [1, 2] Although malaria transmission appears to be declining worldwide as a result of control interventions [2, 3], the 2015 estimation indicates that there are 214 million cases and 438,000 malaria deaths [4]. Malaria is caused by five species of *Plasmodium*: *Plasmodium falciparum, Plasmodium vivax*, *Plasmodium ovale*, *Plasmodium malariae* and *Plasmodium knowlesi* [1]. In China, *P. vivax* and *P. falciparum* are the main malaria parasites, with the former being the most dominant species [5]. *Anopheles sinensis*, *Anopheles minimus*, *Anopheles dirus,* and *Anopheles lesteri* are common malaria vectors in China [6].

Prior to 1949, the annual number of malaria cases in China was estimated to be 30 million. Owing to its substantial public health importance, a Malaria Control Programme was initiated in 1955 [7]. Since then, the malaria burden has greatly declined [8, 9], but it has remained a serious public health problem in China with periodic outbreaks [10]. Following an epidemic peak in 2006 [11], control efforts were consolidated with the formulation of the National Malaria Control Programme (NMCP) in 2006 [12]. Overall, malaria cases have sharply declined with only 14,491 malaria cases reported in 2009 [13]. The National Malaria Elimination Programme (NMEP) was launched in 2010 [12]. Since then, substantial progress has been made. *P. vivax* malaria cases were reduced by 57.7% in one year [14], followed by a decline in geographical coverage [5]. However, *P. falciparum* greatly increased dominating the overall confirmed malaria cases since 2007. The proportion of *P. falciparum* malaria increased from 7.1% in 2009 [13] to 71.2% in 2013 [15]. Areas affected by *P. falciparum* have consistently increased from 17 provinces in 2006 to 20 in 2010, 22 in 2011 [14], and to 30 provinces in 2013 [14–16], involving formerly non-endemic provinces [17].

The distribution of malaria in China shows considerable variation at fine spatial resolution such as county [18, 19]. A better understanding of the spatiotemporal change in disease distribution is crucial for improving control interventions and health resource allocation. Several studies have used the spatial and space–time scan statistics to detect clustering of malaria [18–22] and other public health problems [23] in space and time. These techniques detect disease clusters while adjusting for varying population size among spatial and temporal scales under study. In China, these have been used to identify high-risk areas and periods of malaria in some endemic provinces [18–22, 24, 25]. However, few studies have analysed the spatial and space–time distribution of both *P. vivax* and *P. falciparum* malaria at the national level. The purpose of the present study is to fill this gap in the understanding of the spatial and spatiotemporal distribution malaria in China during 2005–2014.

## Methods

The study was conducted in mainland China, which encompass 31 provinces/autonomous region/municipalities, and more than two thousand county-level divisions. According to the sixth national census in 2010, mainland China has a population of 1.3 billion [26].

### Data sources and its management

Malaria case data between 2005 and 2014 were obtained from the China Information System for Disease Control and Prevention (CISDCP). Malaria is a notifiable disease in China. The laboratory-confirmed and suspected cases are reported to the county-level CDC within one day through an online infectious disease reporting system in use since 2004 [27]. Case investigation and identification are conducted within three days of receiving the case report. Cases are identified according to national standard criteria issued by the Chinese Ministry of Health [28]. Malaria case data includes the county name and associated county code (i.e., identification number), demographic information, date of diagnosis and travel history. These counties are the living addresses for malaria cases. The surveillance-response system covers the whole country which enabled us to conduct a national level study.

Malaria case data, population, and geo-coordinates of each county were linked via county code in R Software version 3.2.2 [29]. As input for the clustering analysis in SaTSan software (version 8.0), a coordinate file, case file and population files were separately generated in text format. To indicate locations of the clusters, the output of the clustering analysis was mapped in ArcGIS software version 10.3.1 [30].

### Spatial autocorrelation analysis

A descriptive analysis was performed to explore county-level annual trends of *P. falciparum* and *P. vivax* malaria cases in China during 2005–2014, and the results were plotted to reveal the annual trend. County level counts of *P. vivax* and *P. falciparum* malaria case for each year were used to test for the spatial distribution of the malaria in China. To explore the overall spatial autocorrelation within-country level local malaria cases, Global Moran’s *I* statistics was applied in ArcGIS software version 10.3.1 [30]. Row standardization of spatial weight was allowed as criteria for identifying neighbouring counties [31]. If counties share a boundary the spatial weight element was assumed to be 1; otherwise it was assumed to be 0. Based on this assumption, Global Moran’s *I* values were calculated for *P. falciparum* and *P. vivax* malaria separately. The value varies between −1.0 and +1.0, where a value close to +1 indicates a tendency for spatial clustering (positive spatial autocorrelation) of areas with similar numbers of malaria cases, whereas −1.0 and 0.0 respectively indicate spatial dispersion (negative spatial autocorrelation) and a random spatial pattern. Global Moran’s *I* tests the null hypothesis that the distribution of malaria in China is random in space [32]. Significance (p < 0.05) of the test statistic suggests that malaria cases are spatially clustered or dispersed. Following this, spatial scan statistics was used to identify locations of the significant cluster (i.e., high-risk areas) [33].

### Spatial and space–time cluster analysis

In this study, two spatial scan statistics were performed: purely spatial cluster analysis and mixed space–time cluster analysis. The purely spatial cluster analysis (with the Poisson model) was performed for *P. vivax* and *P. falciparum* malaria separately in SaTScan Software. These techniques impose circular windows of different sizes to scan for spatial clustering. Kuldorff’s spatial cluster detection statistics [34], was used to scan for counties with high rates of malaria. The maximum spatial cluster size was set to the default of 50% of the population at risk in the circular spatial window [18]. In addition to avoiding pre-selection bias, this enables scanning for clusters of different sizes [34]. The most likely and secondary clusters were not permitted to overlap. The space–time scan statistic was also performed with the Poisson model. The length of time aggregation was set to one year. The maximum temporal cluster sizes to be scanned were also set at the default of 50% of the populationat risk and 50% of the study period, respectively.

## Results

### Overall trend of malaria

A total of 247,540 malaria cases were reported in China during 2005–2014. An annual variation in number of malaria cases was observed with most cases in 2006 (N = 73, 283), comprising 56,258 *P. vivax* and 3113 *P. falciparum* malaria. Overall, *P. vivax* accounted for 75.2% of malaria cases, while 8.7% were *P. falciparum*. 16.2% of all malaria cases reported during this period were unidentified.

**Fig. 1:**
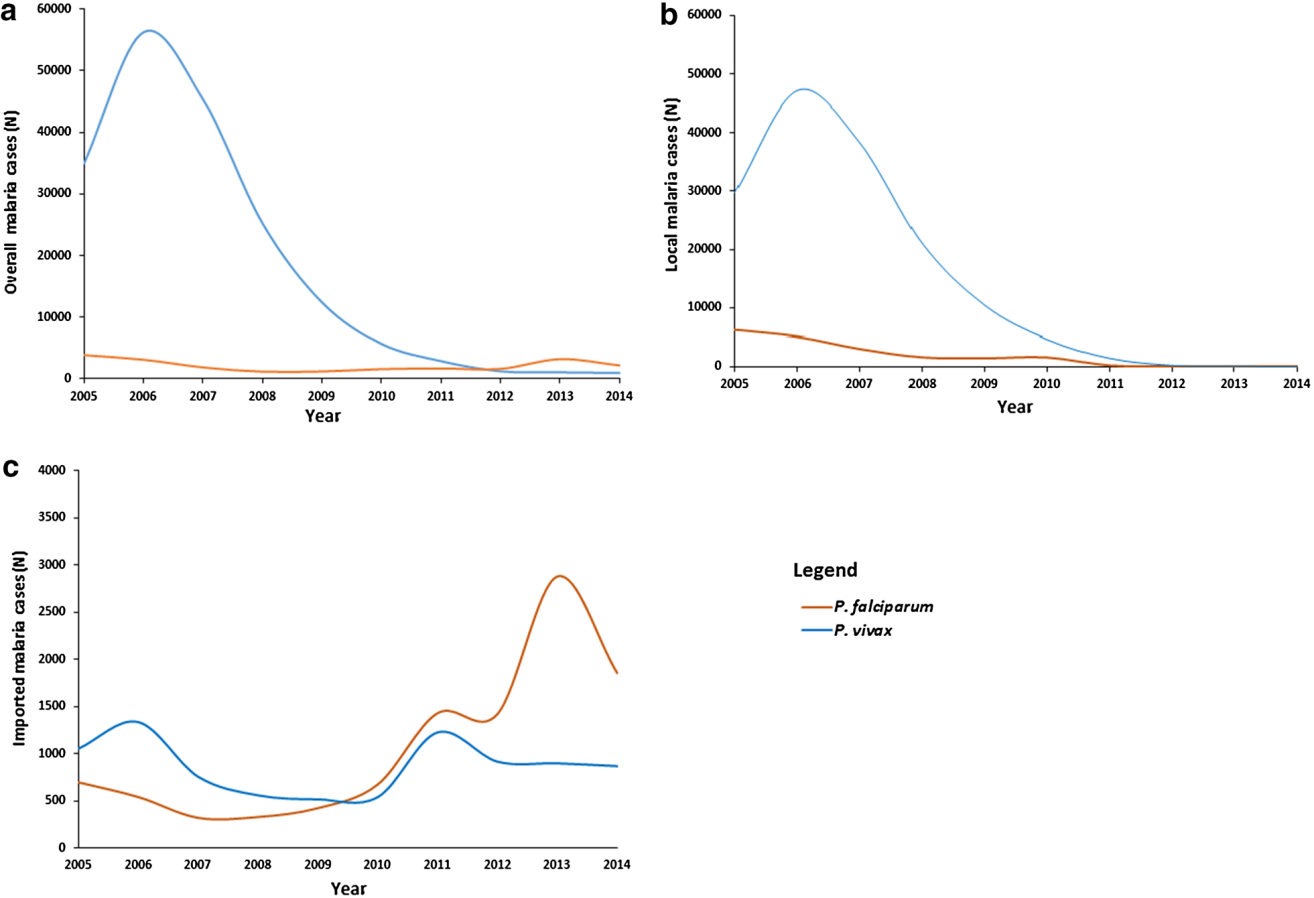
Annual trend of overall, local and imported *P. vivax* and *P. falciparum* malaria in China from 2005 to 2014. **a** Overall trend of *P. vivax* and *P. falciparum* malaria. **b** Annual trends of local *P. vivax* and *P. falciparum* malaria. **c** Annual trends of imported *P. vivax* and *P. falciparum* malaria

A substantial decrease in the yearly number of *vivax* malaria case was observed during the study period (Fig. 1a). The most rapid reduction in the number of *P. vivax* cases occurred after 2006. The number of areas with *P. vivax* cases declined from 791 counties in 2006 to 303 counties in 2014 with an overall reduction of 60.7%. Numbers of *P. falciparum* malaria cases reached a peak in 2005, fell until 2008, then slightly increased, and have dominated since 2012 (Fig. 1a). Consequently, areas with *P. falciparum* greatly increased from 228 counties to 783. Compared to 2005, areas with *P. falciparum* increased by more than 100%.

### Sources of infection

The data from 2005 to 2014 indicated 153, 051 local *P. vivax* and 19, 666 local *P. falciparum* malaria cases. Declining trends of local transmission were observed for both malaria types (Fig. 1b). Local *P. vivax and P. falciparum* malaria transmission declined by 99.79 and 98.42%, respectively. The geographical distribution of the counties with local *P. vivax* and *P. falciparum* during this period was shown in Additional file 1.

In contrast to the decline in local malaria cases, an increasing trend was observed for imported malaria during this study period (Fig. 1c). Imported *P. falciparum* consistently increased from 2005 to 2014, reaching a peak in 2013. However, the imported *P. vivax* didn’t show a significant change over the study period. Regarding the origin of infection, *P. falciparum* was mainly imported from Africa (72.70%, N = 7, 700) and Myanmar (17.7%, N = 1, 878) while the remaining cases were imported from Indonesia (0.69%, N = 73), Cambodia (0.23%, N = 24), Laos (0.19%, N = 20), Pakistan (0.13%, N = 14), India (0.09%, N = 10), Papua New Guinea (PNG) (0.06%, N = 6), and Vietnam (0.06%, N = 6), and. *P. vivax* was mainly imported from Myanmar (N = 5, 613), Oceania and unspecified countries (N = 1, 171) (Additional file 2).

Spatial autocorrelation analysis of annual local malaria cases in China showed the existence of significant overall spatial autocorrelation each year during the study period. The tendency of spatial clustering was observed for both *P. vivax* and *P. falciparum* malaria (Table 1).

**Table 1:** Spatial autocorrelation (Global Moran’s *I*) of *P. vivax* and *P. falciparum* in China during 2005–2014

Year	*P. vivax*			*P. falciparum*		
	*I*	Z	p value	*I*	Z	p value
2005	0.42	20.1	< 0.001	0.11	43.01	<0.001
2006	0.53	21.5	<0.001	0.10	52.19	<0.001
2007	0.49	21.25	<0.001	0.07	42.35	<0.001
2008	0.53	29.24	<0.001	0.08	51.45	<0.001
2009	0.52	24.03	<0.001	0.08	45.56	<0.001
2010	0.34	20.85	<0.001	0.07	38.32	<0.001
2011	0.28	17.97	<0.001	0.05	24.18	<0.001
2012	0.19	14.86	<0.001	0.02	9.42	<0.001
2013	0.09	5.90	<0.001	0.01	1.86	<0.01
2014	0.07	38.22	<0.001	0.03	16.61	<0.001

*I* Moran’s Index, *Z* z-score

### Spatial clustering

**Fig. 2:**
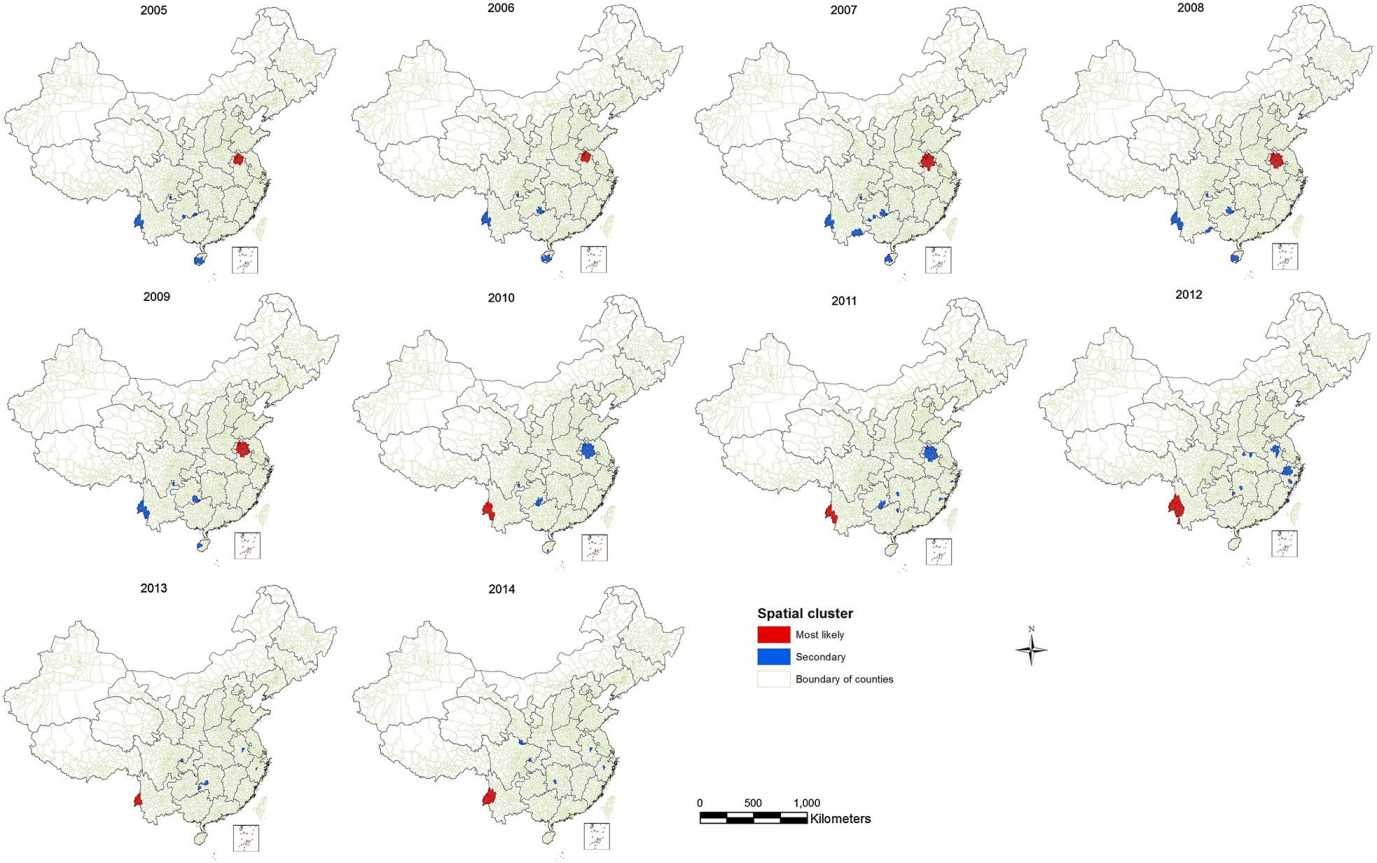
The locations of the detected spatial clusters of *P. vivax* malaria in China from 2005 to 2014

A purely spatial cluster analysis of annual *P. vivax* malaria indicated a non-random distribution of malaria cases in China during 2005–2014. The analysis detected significant spatial clusters (most likely and secondary) each year during the study period. The number of counties per most likely cluster ranged from 7 to 35. The most likely cluster with the highest number of counties was detected in 2007 and that with the lowest number in 2013. The most likely spatial cluster of *P. vivax* included several counties in the Northern Anhui province from 2005 to 2009. Throughout the rest of the study period, the most likely cluster covered bordering counties along the western Yunnan province. The secondary clusters were also identified in many provinces, each year during the study period. Most of these clusters, especially those with a high relative risk were detected in Western Yunnan from 2005 to 2009 and in Northern Anhui until 2011 (Fig. 2). Overall, more than half of the local *P. vivax* cases were reported from the most likely and secondary cluster areas except in 2013, when the clusters contributed to 42.62% of the local *P. vivax* malaria cases reported in the country (Table 2).

**Table 2:** Pure spatial clustering of *P. vivax* cases in China during 2005–2014

Year	Clusters	Latitude	Longitude	N	Observed cases	Expected cases	RR	p value
2005	Most likely	33.68399	116.72312	15	15,241	570.93	48.44	0.001
	Secondary	24.03683	97.80955	22	5590	105.74	63.35	0.001
2006	Most likely	33.68399	116.72312	15	35,053	958.00	97.94	0.001
	Secondary	24.03683	97.80955	23	5595	177.28	34.99	0.001
2007	Most likely	33.21876	116.58207	35	31,436	1543.33	66.53	0.001
	Secondary	24.03683	97.80955	29	3122	143.51	23.32	0.001
2008	Most likely	33.03381	117.03400	32	15,766	717.31	57.44	0.001
	Secondary	24.03683	97.80955	28	2091	109.56	20.73	0.001
2009	Most likely	33.03381	117.03400	32	6533	345.07	39.07	0.001
	Secondary	24.03683	97.80955	21	1561	53.42	33.31	0.001
2010	Most likely	24.03683	97.80955	13	1320	30.02	57.20	0.001
	Secondary	33.03381	117.03400	39	1872	151.63	18.03	0.001
2011	Most likely	24.03683	97.80955	12	799	11.67	95.57	0.001
	Secondary	33.03381	117.03400	39	678	72.60	12.01	0.001
2012	Most likely	24.03683	97.80955	23	516	9.26	101.22	0.001
	Secondary	28.20380	120.13838	22	20	1.06	19.19	0.001
2013	Most likely	24.85646	97.91907	7	289	2.31	183.35	0.001
	Secondary	32.00298	117.56783	9	22	0.76	29.67	0.001
2014	Most likely	25.27506	98.49739	14	324	4.49	113.70	0.001
	Secondary	32.94082	104.77725	7	47	0.19	261.31	0.001

*N* number of counties per cluster, *RR* indicates relative risk for malaria case in the location

**Fig. 3:**
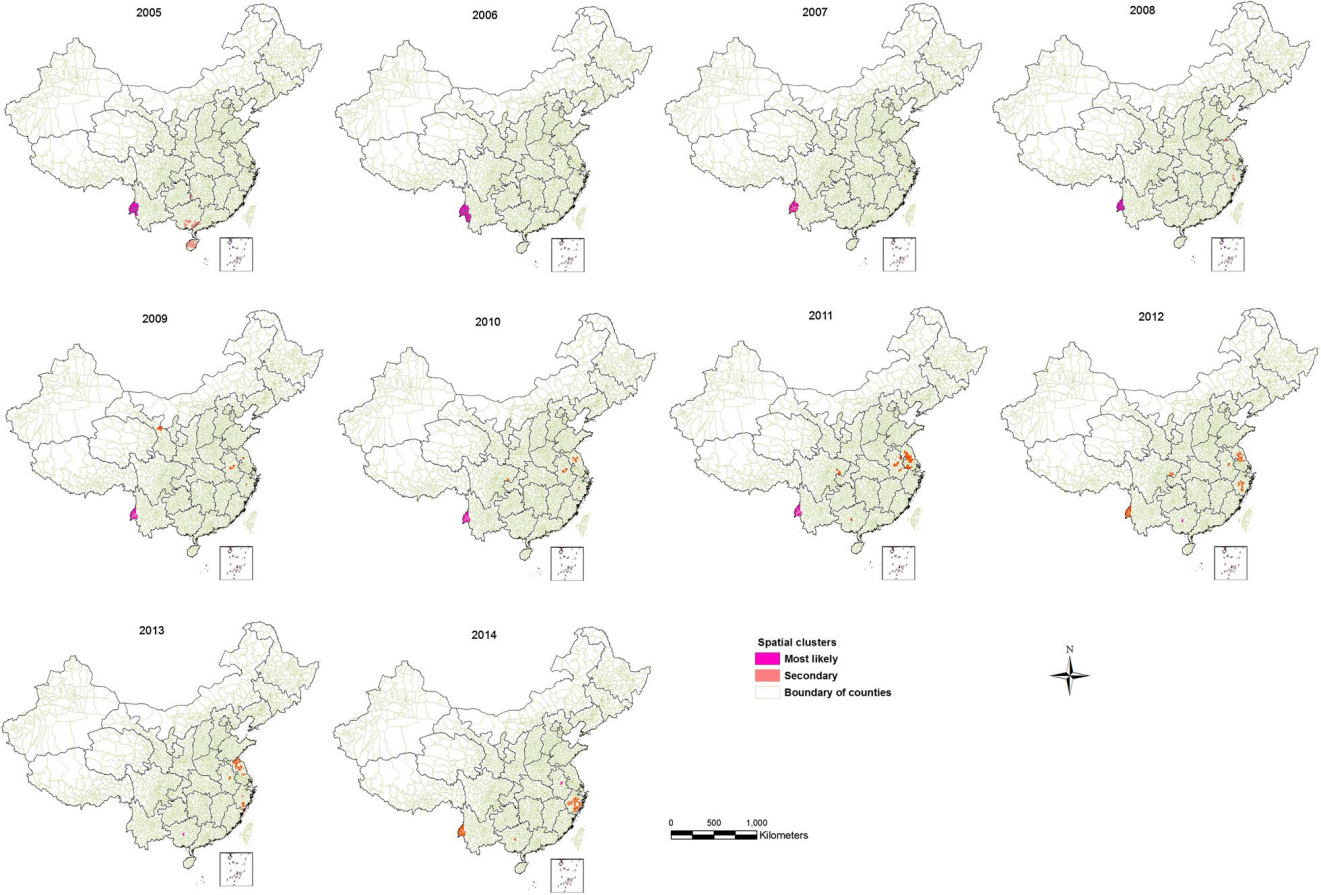
Location of the detected spatial clusters of *P. falciparum* malaria cases in China from 2005 to 2014

The distribution of *P. falciparum* malaria also showed spatial clustering during 2005–2014. From 2005 to 2011, the most likely cluster was consistently detected in western Yunnan province along the China–Myanmar border (Fig. 3). The number of counties per most likely clusters during this period ranged from 7 to 13. The cluster with the highest number of counties (n = 13) was detected in 2006. This cluster contributed to more than half of the local *P. falciparum* malaria cases reported each year from 2005 to 2011 (i.e., 78.52, 77.54, 65.97, 58.22, 55.00, 54.61 and 46.71%, respectively). During 2012–2014, the most likely spatial cluster was detected in the counties of central Guangxi (in 2012 & 2013) and Anhui (in 2014). The most likely spatial cluster detected during this period included a small number of counties. However, 48.90% of the total counties per secondary spatial clusters were detected during 2012–2014. Overall, the spatial cluster areas contributed to more than half (53.2%) of the local *P. falciparum* malaria cases reported during 2012–2014 (Table 3).

**Table 3:** Pure spatial clustering of *P. falciparum* cases in China during 2005–2014

Year	Clusters	Latitude	Longitude	N	Observed cases	Expected cases	RR	p value
2005	Most likely	24.73918	98.31948	10	2545	27.18	335.72	0.001
	Secondary	21.08838	110.32950	25	198	103.24	1.97	0.001
2006	Most likely	24.03683	97.80955	13	2265	28.68	348.14	0.001
2007	Secondary	24.73918	98.31948	9	1118	12.66	249.20	0.001
	Most likely	29.52352	119.90339	1	20	1.55	13.08	0.001
2008	Secondary	24.85646	97.91907	7	590	5.02	255.22	0.001
	Most likely	34.55674	118.78395	4	26	2.85	9.33	0.001
2009	Secondary	24.85646	97.91907	7	508	4.97	192.20	0.001
	Most likely	37.52641	103.33228	8	22	0.92	24.47	0.001
2010	Secondary	24.85646	97.91907	7	529	6.35	133.89	0.001
	Most likely	30.53620	106.41193	11	61	4.63	13.74	0.001
2011	Secondary	24.85646	97.91907	7	213	5.67	45.08	0.001
	Most likely	32.10585	118.76075	38	297	54.25	6.88	0.001
2012	Secondary	23.52086	108.63520	1	112	0.75	167.01	0.001
	Most likely	24.85646	97.91907	31	144	4.39	37.48	0.001
2013	Secondary	23.52086	108.63520	1	955	1.46	1081.9	0.001
	Most likely	32.00298	117.56783	21	134	3.58	39.53	0.001
2014	Secondary	32.00298	117.56783	1	90	1.82	52.79	0.001
	Most likely	23.52086	108.63520	32	56	0.75	77.63	0.001

*N* number of counties per cluster, *RR* indicates relative risk for malaria case in the location

### Space–time clustering

**Fig. 4:**
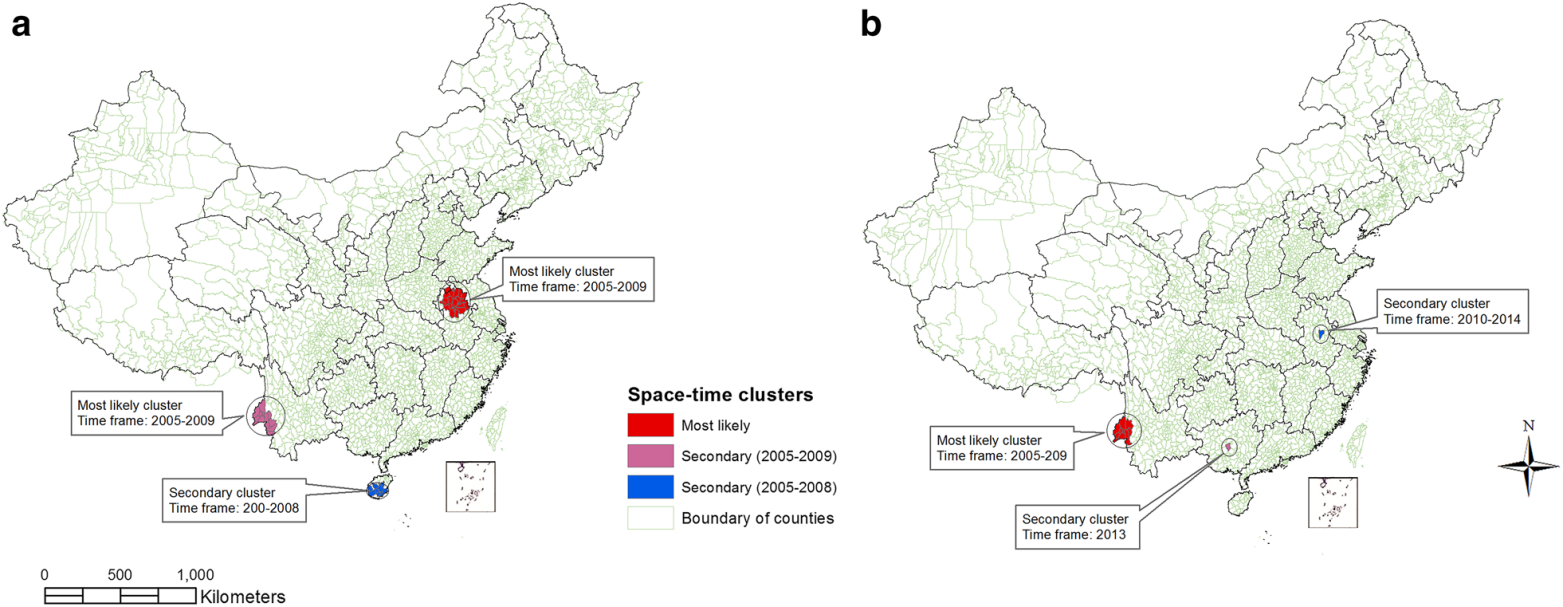
Location of space–time clustering of malaria in China from 2005 to 2014. **a** locations of spatiotemporal clusters of *P. vivax*; **b** locations of spatiotemporal clusters of *P. falciparum*

The space–time scan statistics identified both the most likely and secondary clusters of locations with an elevated risk of *P. vivax* and *P. falciparum* malaria (Table 4). The most likely space–time cluster of *P. vivax* encompassing 32 counties was in a northern part of Anhui province in 2005–2009 (Fig. 4a). A secondary space–time cluster of *P. vivax* encompassing 20 counties was identified in the western part of Yunnan (2005–2009) and Hainan (2005–2008) provinces. For *P. falciparum*, the most likely space–time cluster included 10 counties in the western part of Yunnan province during the same period. A secondary space–time cluster of *P. falciparum* was detected in Anhui, Guangxi, and Zhejiang province from 2012 to 2013 (Fig. 4b).

**Table 4:** Space–time clustering of *P. vivax* and *P. falciparum* malaria in China, 2005–2014

Malaria parasites	Clusters	Latitude	Longitude	Start/end date	N	Observed cases	Exp. case	RR	p value
*P. vivax*	Most likely	24.73918	98.31948	2005/2009	10	7011	67.29	170.70	0.001
	Secondary	23.52086	108.63520	2013/2013	24	955	1.13	895.75	0.001
*P. falciparum*	Most likely	33.03381	117.03400	2005/2009	32	10,829	2538.07	103.88	0.001
	Secondary	24.03683	97.80955	2005/2009	26	18,919	385.80	54.61	0.001

*N* number of counties per cluster, *RR* indicates relative risk for malaria case in the location. *Obs. case* observed number of case in the cluster, *Exp. case* expected number of cases in the cluster

## Discussion

Using a surveillance dataset of 10 years, the present study demonstrated substantial changes occurring with respect to annual trends and the geographical distribution of malaria in China. The annual number of *P. vivax* malaria cases showed a considerable decrease, especially after 2006 when the NMCP was launched [12]. However, the number of *P. falciparum* cases remained relatively stable at a higher level. *P. falciparum* cases malaria peaked in 2005, fell until 2008, then slightly increased, and has dominated cases since 2012. The steady decline in *P. vivax* cases and increase in *P. falciparum* cases was similar to that noted previously [5].

The present study also indicated a shrinking in the geographical distribution of *P. vivax* malaria and a substantial expansion in areas with *P. falciparum*. These findings coincide with previous research [15, 35]. Different interventions have been formulated with the aim of controlling malaria in China. These have been effectively implemented with support from the Global Fund to Fight AIDS, Tuberculosis and Malaria (GFATM) [36]. This may have increased the ability of counties to effectively control local malaria transmission dominated by *P. vivax* [15, 16]. A possible reason for the geographical expansion of *P. falciparum* malaria could be the rising number of overseas imported malaria cases in recent years [14, 37]. As of 2013, 97.9% of the national malaria cases reported came from overseas [15]. Most overseas-imported malaria in recent years was *P. falciparum*. One study conducted in Jiangsu province showed that *P. falciparum* accounted for 79.8% of the total malaria cases imported to the province during 2001–2011 [38]. In recent years, a dramatic increase in overseas investments increased the number of Chinese persons working abroad and paved the way for international travel to and from countries where *P. falciparum* is highly endemic. In 2012, about 0.5 million people left the country for work and 83.2 million for other reasons. Compared to 2010, an increase of 24.6 and 44.9% were observed, respectively [39]. Available evidence indicated that *P. falciparum* was mostly imported via Chinese people returning from Africa [17, 38, 40–44].

Identifying a high-risk area is crucial for spatial targeting of interventions against malaria transmission. Spatial cluster analysis identified most likely clusters of *P. vivax* malaria located in North Anhui province from 2005 to 2009. The area where most likely clusters of *P. vivax* were identified in this study was similar to the previous transmission foci detected in the northern part of Anhui province [18], where *An. sinensis* is a principal vector. A study suggested that increased vector capacity of *An. sinensis* related with the mosquito host reduction (livestock), and human behavioural change contributed to *P. vivax* malaria outbreak in Huaiyuan county of Anhui province [45]. This area, especially north of the Huai River, is one of the high-risk areas with unstable malaria transmission [46], possibly due to environmental conditions associated with geographical location [11, 14, 18]. Malaria transmission in this province has been an important issue in China, responsible for the outbreak in 2006 which was dominated by *P. vivax* [11]. From 2006 to 2009, Anhui had been the number one province in China in terms of number of malaria cases [11, 13, 47].

The present study also identified most likely spatial clusters of *P. vivax* malaria in western Yunnan province, along the China–Myanmar border after 2009. The cluster persisted for five years (2010–2014), contributing more than half the total number of *P. vivax* cases reported each year. Although the high-risk area identified in this area agrees with previous studies [20, 48, 49], a shift of the geographical location from Anhui to Yunnan province after 2009 is new. The reduction in Anhui may be explained by intensive malaria control in the central China provinces [50]. Since the initiation of the NMEP in 2010 [12, 51], national malaria, especially local cases greatly declined [8, 15]. One study showed that overall malaria cases in Anhui province decreased by 65.5% (in 2011) compared to those in 2010 [9]. The total number of *P. vivax* cases in the country, therefore, decreased by 57.7% in one year, with most of the local cases in Yunnan province [16]. The province remained endemic, ranking first in the country in terms of an overall number of malaria cases [8, 9, 15, 52], particularly *P. vivax* [16]. For example, 73.1% (171/234) of the national *P. vivax* malaria cases in 2012 were contributed by Yunnan [16], and *P. vivax* malaria appeared to be dominant along the China–Myanmar border [53]. Malaria transmission in this area is a major concern in the disease elimination stage. This could be attributed to environmental conditions conducive for transmission and efficiency of the dominant *An. minimus* vectors in this area [17]. Unlike *An. sinensis* [54, 55], *An. minimus* shows a strong attraction to human than other hosts [56] and has a high human blood index [57] which has an implication for vector control interventions even though *An. minimus* is endophilic, endophagic, and susceptible to insecticides [55, 58]. Human behavioural factors [59] could be another reason for malaria transmission and control in this area. For example, some counties of Yunnan province along the international border have been recognized as high-risk areas in China because of sharing a boundary with malaria endemic countries which put them at risk of reintroduction [20, 60]. One study revealed that imported *P. vivax* to China had increased between 2004 and 2012, most of which were from malaria endemic countries of South East Asia (Myanmar, Cambodia and Laos) [16]. 59.3% of the total *P. vivax* malaria cases imported from South East Asia (n = 697) were introduced from Myanmar, and 56.7% (n = 6832) of the total introduced *P. vivax* cases (n = 12, 060) were in Yunnan [17]. However, other evidence shows lack of significant association between travel to Myanmar and transmission of *P. vivax* along the China–Myanmar border [53]. Residents in this area have a relatively low educational level, limited knowledge of malaria transmission and utilization of personal protection, especially during outdoor activities [61, 62], and exhibit poor treatment-seeking behaviour [62]. These factors could contribute to sustained malaria transmission in this area [17].

Similarly, the current study identified the most likely spatial clusters of *P. falciparum* malaria in the western Yunnan province along the China*–*Myanmar border every year from 2005 to 2011. This is consistent with previous studies [20, 48, 49]. This is the only area where the local *P. falciparum* cases were reported by several studies [9, 15]. It implies that these counties are at high-risk of achieving stable *falciparum* malaria transmission. Most of the national *P. falciparum* cases during 2005–2011 were reported from Yunnan province, although counties were not frequently specified [9, 11, 47, 52]. In addition to behavioural and lifestyle factors for malaria transmission [61], travel to Myanmar was significantly associated with acquiring *P. falciparum* infection, indicating cross-border movement is a key factor for the stable transmission of *P. falciparum* in the China–Myanmar border [17, 53, 61, 62]. However, 84.5% of the imported *P. falciparum* cases were imported from Africa. Climatic factors have also been found to be an important factor for malaria transmission in this area [48]. According to one study [20], temperature was significantly associated with vivax malaria in clustered areas of Yunnan province. Further research is required to better understand the importance of climatic factors in the spatial distribution of malaria in China.

The present study also identified high-risk areas for *P. falciparum* in central Anhui province from 2013 to 2014. This foci (Feidong county) is different from the previously identified high-risk area in the Northern Anhui province [18], indicating spatial variation in foci of *P. falciparum* cases, was and its less importance in this area than *P. vivax* [11]. In the present study, the most likely spatial cluster of *P. falciparum* was not detected in Anhui province until 2012. However, a spatial cluster of *P. vivax* was observed consistently in the northern Anhui province from 2005 to 2009. This result verified that *P. falciparum* played a relatively insignificant role in the previously identified high-risk area in Anhui province [18].

The high-risk area for *P. falciparum* malaria shifted from Yunnan to Anhui province, and very large secondary clusters were detected in some counties of the northern and eastern provinces, especially after 2011. This could be attributed to the increased proportion of overseas imported malaria from the parasite endemic countries in recent years [14, 46]. *P. falciparum* dominates overseas imported malaria cases, which are distributed in different parts of China, including non-endemic provinces. Nevertheless, the study of disease clustering focused only on local malaria cases. Spatial variations among *P. vivax* and *P. falciparum* malaria followed different patterns indicating differences in the biological features of parasites, which might have facilitated their transmission. Climatic factors are associated with an increased risk of malaria because of their impact on vector activities and the parasite incubation period [63]. Compared to *P. vivax*, *P. falciparum* requires a slightly higher temperature for parasite development. The minimum threshold temperature for *P. falciparum* and *P. vivax* are approximately 18 and 15 °C, respectively [64], indicating increased opportunity for *P. falciparum* to spread to previously cooler areas, following global climate change. Further studies are required to fully understand the risk factors driving spatial shifting and geographic expansion of *P. falciparum* across mainland China.

The most likely space–time cluster of *P. vivax* malaria was detected in the northern Anhui province. These areas coincided with results of the purely spatial analysis in this study as well those of previous studies [18]. The time frame for all significant space–time clusters of *P. vivax* malaria was 2005–2009. This implies a declining burden of *P. vivax* malaria in China since 2010. Control interventions, especially those following the establishment of the Chinese NMEP [12, 51] are a likely factor in this substantial reduction of this malaria.

For *P. falciparum*, the most likely space–time cluster was detected in western Yunnan province during 2005–2009. Although this is consistent with the previous study [65], the secondary space–time cluster was scattered mostly in the eastern and north-eastern provinces after 2012 (Fig. 4b). This could imply the spreading of *P. falciparum* malaria to previously non-endemic areas, probably due to an increased number of overseas-imported *P. falciparum* [8, 15, 35]. Although an imported case can be distributed randomly to any provinces, the present study showed a greater expansion of *P. falciparum* to the east and northeast of China than to any other parts of the country. A better understanding of the direction of malaria expansion or spatial change and underlying risk factors for the malaria transmission in these formerly non-endemic areas is important for the malaria elimination goal of China.

This study is the first to identify the spatial and spatiotemporal distribution of *P. vivax* and *P. falciparum* at the national level in China. A separate analysis was conducted for both important malaria parasites in the country. The study identified high-risk areas and the spatial extent of both *P. vivax* and *P. falciparum*. Spatial and space–time scan statistics were performed using SaTScan software [66]. These techniques were designed particularly to perform spatial clustering of disease or health related events, and to test the statistical significance of clustering under the null hypothesis of a random distribution of the diseases in space, time and space–time [66, 67]. These techniques are most effective at identifying disease clusters [68], and have been widely used in fields of epidemiology for similar purpose [18, 19, 21, 24, 25].

Although purely spatial scan statistics are most effective at identifying a cluster of malaria with a circular shape [68], the spatial distribution of the disease may not always assume this shape and some irregularly shaped clusters might have been undetected.

## Conclusion

Overall, the spatial and space–time cluster detection statistics identified a high-risk areas for both *P. vivax* and *P. falciparum* malaria. The counties in the clusters should be given priority in the control programs, and for further operational research. Both malaria types showed significant spatial and spatiotemporal variations. Contrary to *P. vivax*, the high-risk areas for *P. falciparum* malaria shifted from southwest to east of China. Thus, further studies are required to examine spatial changes in the risk of malaria transmission and identify the underlying causes of elevated risk in emerging high-risk areas.

## Additional files

**Additional file 1.** Distribution maps of imported malaria cases.

**Additional file 2.** Number of malaria cases imported from other countries, 2005–2014.

## Abbreviations

CISDCP: China information system for disease control and prevention
NMCP: National Malaria Control Programme
NMEP: National Malaria Elimination Programme
CDC: Center for Disease Control and Prevention
I: Moran’s index
Z: z-score
RR: indicates relative risk
AIDS: acquired human immuno deficiency syndrome
