# Supplementary figures and images for: Spatial and space–time distribution of Plasmodium vivax and Plasmodium falciparum malaria in China, 2005–2014
Source: Malar J. 2016 Dec 19;15:595. doi: 10.1186/s12936-016-1646-2 (PMC5168843; doi:10.1186/s12936-016-1646-2)

Additional file 1: Distribution maps of imported malaria cases

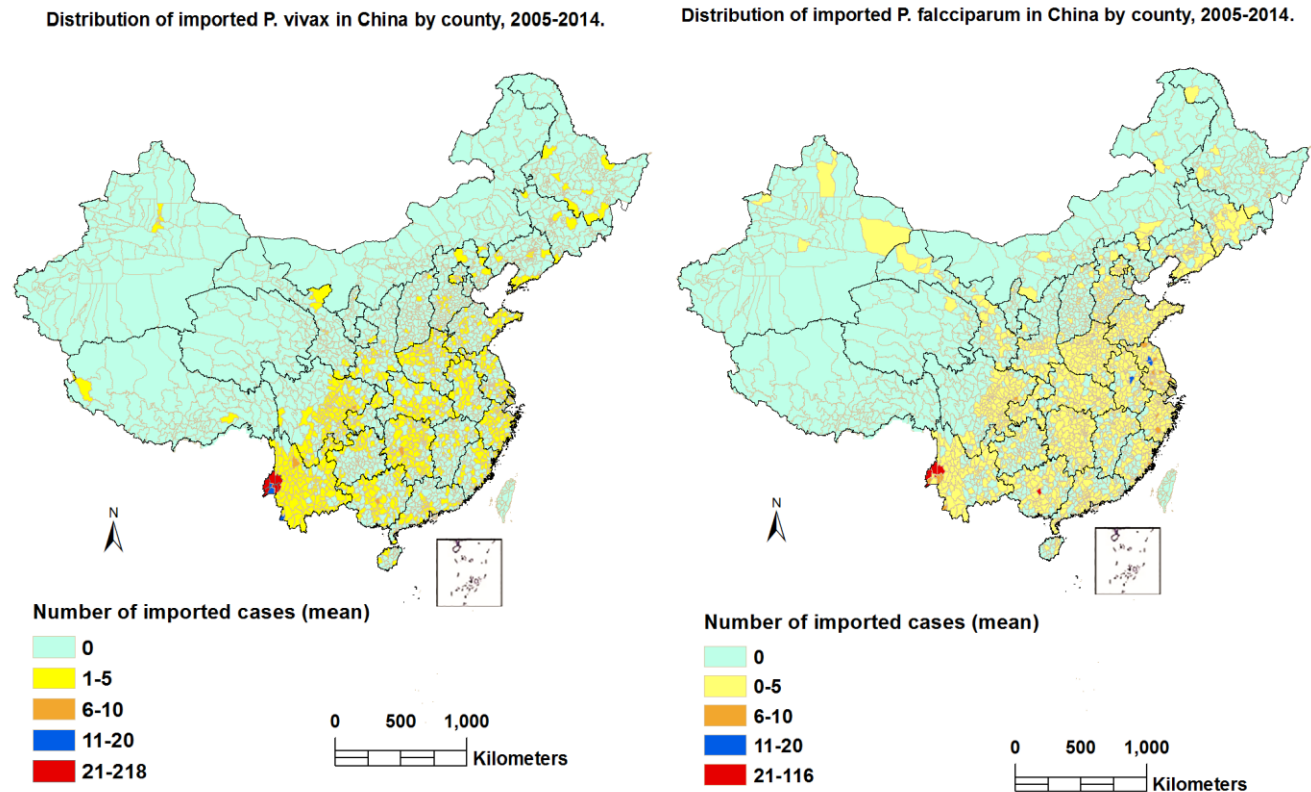

Supplement: Supplementary file 1 — Additional file 1. Distribution maps of imported malaria cases. [file 12936_2016_1646_MOESM1_ESM.pdf]
