# Supplementary material for: Spatial and space–time distribution of Plasmodium vivax and Plasmodium falciparum malaria in China, 2005–2014
Source: Malar J. 2016 Dec 19;15:595. doi: 10.1186/s12936-016-1646-2 (PMC5168843; doi:10.1186/s12936-016-1646-2)
Supplement: Supplementary file 2 — Additional file 2. Number of malaria cases imported from other countries, 2005–2014. [file 12936_2016_1646_MOESM2_ESM.docx]

**Additional file 2: Number of malaria cases imported from other countries, 2005-2014**

| Country of origin | *P. vivax* | *P. falciparum* | Total |
| --- | --- | --- | --- |
| Afghanistan | 2 | 1 | 3 |
| Africa | 1,192 | 7,700 | 8,892 |
| Bangladesh | 1 | 2 | 3 |
| Brazil | 1 | - | 1 |
| Cambodia | 194 | 24 | 218 |
| Canada | - | 1 | 1 |
| Dubai | - | 1 | 1 |
| East Timor | 5 | 7 | 12 |
| India | 76 | 10 | 86 |
| Indonesia | 211 | 73 | 284 |
| Israel | - | 1 | 1 |
| Korea | 3 | - | 3 |
| Laos | 98 | 20 | 118 |
| Malaysia | 5 | 2 | 7 |
| Myanmar | 5,613 | 1,878 | 7,491 |
| Nepal | 1 | - | 1 |
| North Korea | 2 | - | 2 |
| Pakistan | 86 | 14 | 100 |
| PNG | 10 | 6 | 16 |
| Saud Arabia | - | 1 | 1 |
| Seri Lanka | - | 1 | 1 |
| Solomon Islands | 2 | - | 2 |
| South Korea | 3 | - | 3 |
| Thailand | 7 | 3 | 10 |
| Unknown | 1,171 | 834 | 2,005 |
| Vietnam | 4 | 6 | 10 |
| Total | 8,687 | 10,585 | 19,272 |
